# Trans-presentation of IL-15 modulates STAT5 activation and Bcl-6 expression in T_H_1 cells

**DOI:** 10.1038/srep15722

**Published:** 2015-10-26

**Authors:** Ian D. Cooley, Kaitlin A. Read, Kenneth J. Oestreich

**Affiliations:** 1 Virginia Tech Carilion Research Institute, Roanoke, VA; USA; 2 Department of Biomedical Sciences and Pathobiology, Virginia-Maryland Regional College of Veterinary Medicine, Virginia Tech, Blacksburg, VA; USA; 3 Virginia Tech Carilion School of Medicine, Roanoke, VA; USA

## Abstract

During infection, naïve CD4^+^ T helper cells differentiate into specialized effector subsets based upon environmental signals propagated by the cytokine milieu. Recently, this paradigm has been complicated by the demonstration that alterations in the cytokine environment can result in varying degrees of plasticity between effector T helper cell populations. Therefore, elucidation of the mechanisms by which cytokines regulate T helper cell differentiation decisions is increasingly important. The gamma common cytokine IL-15 is currently undergoing clinical trials for the treatment of malignancies, due to its well-established role in the regulation of natural killer and CD8^+^ T cell immune responses. However, the effect of IL-15 signaling on CD4^+^ T cell activity is incompletely understood. One mechanism by which IL-15 activity is conferred is through trans-presentation via the IL-15 receptor alpha subunit. Here, we demonstrate that differentiated T_H_1 cells are responsive to trans-presented IL-15. Importantly, while trans-presentation of IL-15 results in STAT5 activation and maintenance of the T_H_1 gene program, IL-15 treatment alone allows for increased Bcl-6 expression and the upregulation of a T_FH_-like profile. Collectively, these findings describe a novel role for IL-15 in the modulation of CD4^+^ T cell responses and provide valuable insight for the use of IL-15 in immunotherapeutic approaches.

An effective immune response requires the orchestrated activity of a cohort of immune cell types. At the center of this coordinated response are CD4^+^ T helper cells, which provide an instrumental link between the innate and adaptive arms of the immune system. These include T helper 1 (T_H_1), T_H_2, T_H_17, and T follicular helper (T_FH_) cell populations, all of which arise from naïve CD4^+^ T cell progenitors^1^,^2^,^3^,^4^,^5^. The development of each of these effector T helper cell subpopulations is dictated by the expression of specific transcription factors, termed lineage-defining factors, the expression and activity of which are dependent upon the cytokine environment present during the activation of the naïve CD4^+^ T cell^6^,^7^,^8^,^9^,^10^,^11^,^12^. Importantly, each of these populations is responsible for performing unique effector functions that range from the cell-mediated release of inflammatory cytokines during pathogenic infection to aiding in the production of humoral immunity. Despite these distinct roles, numerous studies have recently demonstrated that flexibility, or plasticity, between effector T helper cell populations can occur in response to alterations in the cytokine environment^2^,^13^,^14^,^15^,^16^. Based in part upon these insights, novel sets of immunotherapeutic tools are being developed in an effort to manipulate the immune response and capitalize on the flexible nature of T helper cell populations^17^,^18^,^19^,^20^,^21^. As such, it is increasingly important to understand how changes to the cytokine environment effect not only the initial differentiation of activated naïve CD4^+^ T cells, but also the flexibility of established effector T helper cell populations.

The cytokines IL-2 and IL-15 are well-established regulators of immune cell development and function^22^,^23^,^24^. Both IL-2 and IL-15 signal through hetero-trimeric receptors, which are made up of the gamma common (γc) and IL-2Rβ subunits, as well as specificity-conferring IL-2Rα and IL-15Rα subunits, respectively^19^,^22^. Like IL-2, IL-15 signals through intracellular domains of the γc and IL-2Rβ subunits, which employ the janus kinase 1 (Jak1) and Jak3 kinases to phosphorylate and activate the transcription factor STAT5^19^,^22^. However, IL-15 is capable of signaling through a unique “trans-presentation” mechanism, which involves the expression of IL-15Rα in complex with IL-15 on the surface of a presenting cell^24^,^25^,^26^,^27^,^28^. This complex interacts with the remaining two thirds of the receptor on a target cell, resulting in the propagation of IL-15 signaling within that cell. Trans-presentation has been shown to increase the affinity of IL-15 for the γc and IL-2Rβ receptor subunits, leading to more robust signaling as compared to IL-15 alone^29^,^30^,^31^.

The role of IL-15 in the stimulation of CD8^+^ cytotoxic T and natural killer (NK) cell populations is well-characterized and has provided strong pre-clinical support for its use in cancer and vaccine immunotherapeutic approaches^24^,^27^,^32^,^33^. Currently, IL-15 is undergoing Phase I/II Clinical trials for the treatment of malignant melanoma and renal cell carcinoma^32^,^33^,^34^. However, the effects of IL-15, particularly in the context of trans-presentation, on CD4^+^ T cell differentiation and effector function are incompletely understood. Therefore, an increased understanding of the effects of IL-15 signaling on the diverse repertoire of CD4^+^ T cell gene programs is a critical component in not only understanding how this cytokine may affect the natural immune response, but also in helping to unravel its therapeutic potential.

In the present study, we investigated the effect of IL-15 signaling on CD4^+^ T_H_1 differentiation, using a well-established model of T_H_1 cell generation^35^,^36^. We demonstrate that exposure of T_H_1 cells to trans-presented IL-15, but not to IL-15 alone, favors the T_H_1 gene program. Mechanistically, increased STAT5-activation via trans-presented IL-15 was associated with the expression of T_H_1-associated genes including *Tbx21*, *Ifng*, *Prdm1*, *Il12ra* and *Il12rb*, as well as enhanced IL-12-dependent STAT4 phosphorylation. Strikingly, exposure of T_H_1 cells to IL-15 in the absence IL-15Rα resulted in decreased STAT5 activation and increased expression of the T_FH_ lineage defining transcription factor Bcl-6. Elevated Bcl-6 expression levels correlated with a decrease in Blimp-1 expression and the upregulation of a T_FH_-like gene program including *Cxcr5*, *Il6ra*, *Tnfsf8*, and *Sh2d1a*. Collectively, these results describe previously unappreciated effects of IL-15 signaling on the differential expression of T_H_1 and T_FH_ gene profiles, and provide added insight into the functional properties of this immunotherapeutic cytokine.

## Results

### Exposure to IL-15 alone is insufficient to maintain expression of the T_H_1 gene program

**Figure 1:**
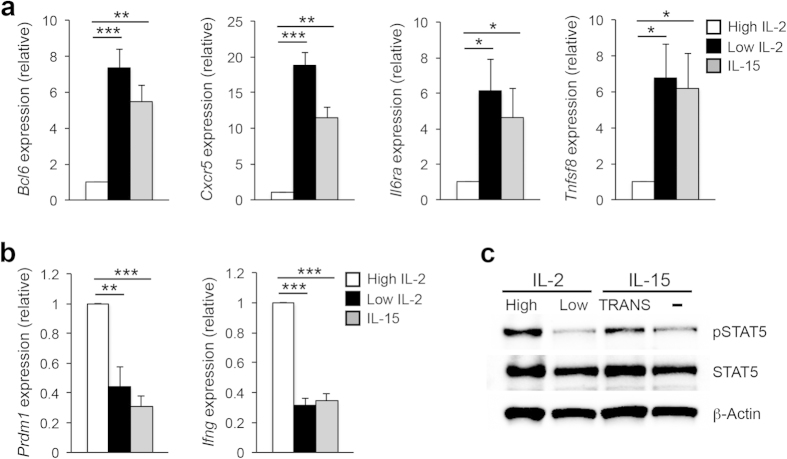
IL-15 treatment alone is insufficient to activate STAT5 and the T_H_1 program. (**a,b**) Primary CD4^+^ T cells were isolated from C57BL/6 mice and stimulated on plate-bound αCD3 and αCD28 for 3 days under T_H_1-polarizing conditions. On day 3, cells were split and cultured with either high IL-2 (250 U/ml), low IL-2 (10 U/ml), or IL-15 (15 ng/ml). Two days following cytokine treatment, RNA was isolated to determine transcript levels for the indicated T_FH_ and T_H_1-associated genes. Samples were normalized to *Rps18* as a control. Data are presented relative to the high IL-2-treated sample. Three (**a,b**) independent experiments were performed with the error bars representing SEM. *P < 0.05, **P < 0.01, ***P < 0.001 (unpaired Student’s *t*-test). (**c**) Cells were treated and harvested as in “a” with the exception that an additional sample was treated with 15 ng/ml of trans-presented IL-15 (IL-15, TRANS). Following cell isolation, an immunoblot assay was performed to assess STAT5 activation levels. Total STAT5 and β-Actin are shown as controls. The image shown is representative of three independent experiments performed.

It has become increasingly evident that mature effector CD4^+^ T cells are responsive to alterations in environmental stimuli in the form of cytokines^13^,^14^,^15^,^37^,^38^,^39^. The adaptability of effector T helper cell populations allows these cells to respond to the environment in real-time, and is particularly important in clinical applications where the use of cytokines or cytokine neutralizing antibodies is employed to promote or abrogate specific immune responses. For example, previous work has demonstrated that signaling through the gamma common (γc) cytokine IL-2 is indispensable for the promotion and maintenance of the T_H_1 cell fate while, conversely, the withdrawal of IL-2 results in a loss of T_H_1 cell identity and the up-regulation of a T_FH_-like gene expression profile^36^,^40^. Interestingly, a second γc cytokine, IL-15, is currently undergoing Phase I/II clinical trials for the treatment of many types of cancer^33^,^34^,^41^. As the downstream signaling events of IL-15 are similar to those of IL-2, we hypothesized that IL-15 may maintain the T_H_1 profile at the expense of T_FH_ gene expression. To begin to test this hypothesis, we examined whether IL-15 treatment alone would be sufficient to both promote T_H_1 gene expression patterns and repress the induction of the T_FH_ gene program following IL-2 withdrawal from differentiated T_H_1 cells. As with our previous study, cells exposed to high IL-2 environments repressed the T_FH_ gene program and maintained T_H_1 expression patterns while, conversely, IL-2 withdrawal from T_H_1 cells resulted in a significant increase in *Bcl6* expression and the induction of a T_FH_-like profile (Fig. 1a,b)^36^. Interestingly, treatment with IL-15 similarly resulted in a significant increase in *Bcl6* expression compared to that observed in high IL-2 cells (Fig. 1a). Importantly, the increase in *Bcl6* expression was associated with an increase in the T_FH_-associated genes *Cxcr5*, *Il6ra*, and *Tnfsf8*. Furthermore, while the T_FH_-associated gene program was upregulated, the expression of the T_H_1 genes *Ifng* and *Prdm1* was decreased (Fig. 1b). One possibility that we considered was that at day 3 (the time-point of IL-15 administration), our *ex vivo* generated T_H_1 cells were not fully committed to the T_H_1 phenotype and therefore maintained a higher degree of flexibility with alternative T helper cell identities. To examine this possibility, we performed a similar experiment, but added IL-15 on day 5 of the T_H_1 culture (rather than day 3). Importantly, a similar induction of the T_FH_-like profile was observed with T_H_1 cells exposed to standard (3 days) or extended (5 days) T_H_1 culturing conditions (Supplementary Fig. S1). Collectively, these results indicate that IL-15 treatment alone is insufficient to support T_H_1 gene expression patterns and instead results in the upregulation of Bcl-6 expression and a T_FH_-like program.

A key finding from our previous work was that the IL-2-dependent phosphorylation status of STAT5 inversely correlated with the expression of Bcl-6^36^. As IL-15 also signals via the activation of STAT5, we hypothesized that IL-15 treatment alone may not be sufficient to phosphorylate STAT5, thus resulting in increased Bcl-6 expression. To test this possibility, we performed an immunoblot analysis to assess the degree of STAT5 phosphorylation in the high IL-2-, low IL-2-, and IL-15-treated T_H_1 cells. While high IL-2 cells displayed elevated levels of STAT5 phosphorylation (pSTAT5), there was very little STAT5 activation in either the low IL-2- or IL-15-treated populations (Fig. 1c). It has been demonstrated that IL-15 is capable of signaling through a unique “trans-presentation” mechanism, which involves the expression of IL-15Rα in complex with IL-15 on the surface of a second presenting cell. Thus, although CD4^+^ T cells have variable expression of IL-15Rα, this suggests that IL-15Rα expression is dispensable for IL-15-responsiveness as trans-presentation provides IL-15Rα in the form of a presenting complex^27^. Therefore, we sought to determine whether T_H_1 cells would display enhanced STAT5 activation in response to trans-presented IL-15. Indeed, we observed increased STAT5 phosphorylation upon exposure of T_H_1 cells to IL-15 in complex with a recombinant IL-15Rα (IL-15_Trans_) (Fig. 1c). Collectively, these data suggest that IL-15 signaling is a potential differential regulator of the opposing T_H_1 and T_FH_ gene programs.

### IL-15 signaling via trans-presentation represses Bcl-6 expression in T_H_1 cells

**Figure 2:**
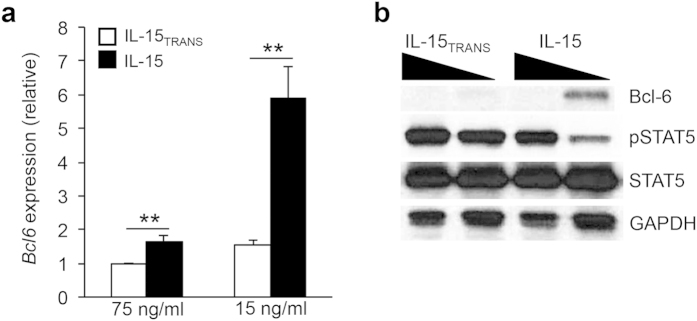
IL-15 signaling represses the expression of Bcl-6. (**a**) Primary CD4^+^ T cells were isolated from C57BL/6 mice and stimulated on plate-bound αCD3 and αCD28 for 3 days under T_H_1-polarizing conditions. On day 3, cells were split and cultured with either IL-15_TRANS_ or IL-15 (75 ng/ml or 15 ng/ml). Two days following cytokine treatment, RNA was isolated to determine the transcript level for *Bcl6*. Samples were normalized to an *Rps18* control and data are represented relative to the IL-15_TRANS_ (75 ng/ml) sample. Five independent experiments were performed with the error bars representing SEM. **P < 0.01 (unpaired Student’s *t*-test). (**b**) Samples were treated and harvested as in “a”. Two days following cytokine treatment, cells were harvested for each condition and an immunoblot analysis was carried out to determine the effect of IL-15 signaling on Bcl-6 expression and STAT5 activation. Total STAT5 and β-Actin are shown as controls. The displayed image is representative of three independent experiments performed.

The demonstration that trans-presentation of IL-15 results in increased STAT5 activation prompted us to more thoroughly explore the effect of IL-15 signaling on the expression of the T_FH_-lineage defining transcription factor Bcl-6. We exposed T_H_1 cells to two different concentrations of IL-15 (75 ng/ml and 15 ng/ml) either alone or in the trans-presented form. Interestingly, upon exposure of cells to a high environmental concentration of IL-15, *Bcl6* expression remained relatively low (Fig. 2a). Conversely, a significant increase in *Bcl6* expression was observed when T_H_1 cells were exposed to a low concentration of IL-15 alone. Importantly, there was a ~6 fold decrease in *Bcl6* expression when the cells were exposed to the same low concentration of IL-15 in the trans-presented form (Fig. 2a). To determine whether Bcl-6 protein expression was consistent with the observed changes in transcript, we performed an immunoblot analysis. Indeed, while there was an increase in Bcl-6 expression at the low IL-15 concentration, we did not observe an induction of Bcl-6 expression in the trans-presented IL-15 sample (Fig. 2b). Importantly, the increase in Bcl-6 expression inversely correlated with the level of IL-15-dependent STAT5 phosphorylation. Collectively, these data indicate that differentiated CD4^+^ T_H_1 cells are capable of responding to IL-15 signaling, particularly during trans-presentation, and that increased IL-15 signaling results in the repression of Bcl-6 expression.

### IL-15 signaling regulates the balance of the transcriptional repressors Bcl-6 and Blimp-1

**Figure 3:**
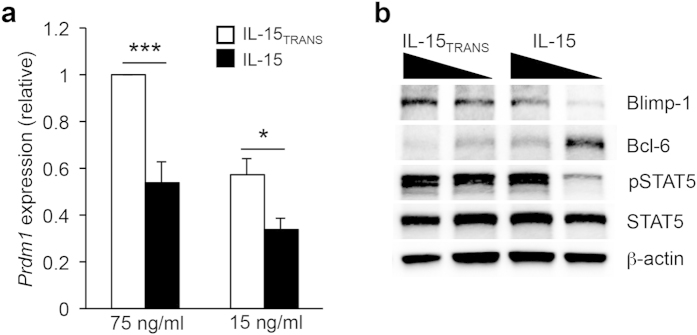
IL-15 trans-presentation maintains Blimp-1 expression. (**a**) Primary CD4^+^ T cells were cultured as described in Fig. 2. Two days following the indicated IL-15 cytokine treatment, cells were harvested for each condition and RNA was isolated to determine transcript levels for *Prdm1* under IL-15 or IL-15_TRANS_ (75 ng/mL or 15 ng/mL) conditions as indicated. Samples were normalized to *Rps18* as a control. Graphical representations are normalized to the IL-15_TRANS_ (75 ng/ml) sample. (**b**) An immunoblot analysis was performed to examine the effect of IL-15 signaling on Blimp-1, Bcl-6, p-STAT5, and STAT5 expression. β-Actin was monitored to ensure equal protein loading. For (**a**), figures are representative of five independent experiments. Error bars represent SEM. *P < 0.05, ***P < 0.001 (unpaired Student’s *t*-test). For (**b**), the image is representative of three independent experiments.

There is a well-established antagonistic relationship between the transcriptional repressors Bcl-6 and Blimp-1 (encoded by the gene *Prdm1*) that guides a number of B and T lymphocyte developmental stages^42^,^43^. For example, previous work from our lab and that of others has demonstrated that Blimp-1 antagonizes T_FH_ cell differentiation^9^,^44^. In the context of T_H_1 cell development, Blimp-1 inhibits the T_FH_ program by directly repressing T_FH_-associated genes including *Cxcr5*, *Il6ra*, and *Tnfsf8*. Therefore, we hypothesized that IL-15 signaling, similar to that of IL-2, may differentially regulate the expression of these opposing transcriptional regulators and ultimately dictate the establishment of a T_H_1 or T_FH_ gene program. Indeed, T_H_1 cells exposed to trans-presented IL-15 maintained elevated expression of *Prdm1*, and importantly, increased Blimp-1 protein expression was also observed with trans-presented IL-15 regardless of high or low environmental concentrations of the cytokine (Fig. 3a,b). Conversely, IL-15 cytokine treatment alone resulted in a decrease in Blimp-1 expression. Importantly, the reduction in Blimp-1 expression was coincident with a decrease in STAT5 phosphorylation, and a corresponding increase in Bcl-6 expression (Fig. 3b).

### Strength of IL-15 signaling differentially regulates T_H_1 and T_FH_ gene expression patterns

**Figure 4:**
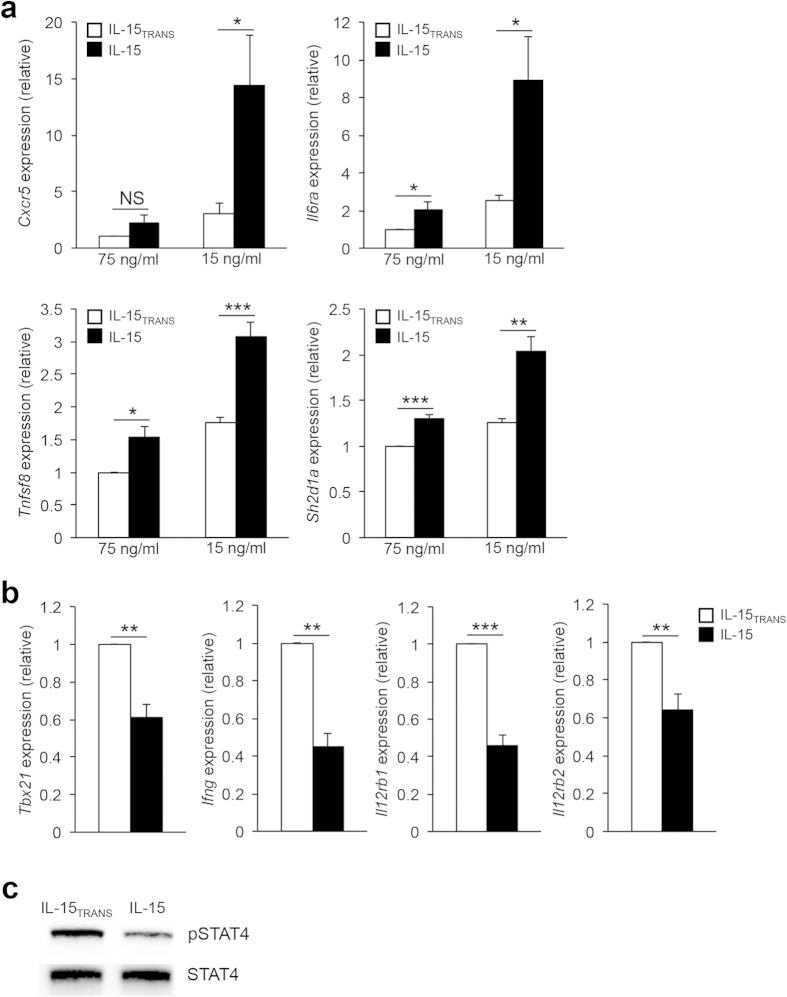
IL-15 signaling regulates the expression of T_FH_-associated genes. (**a**) Primary CD4^+^ T cells were cultured as described in Fig. 2. RNA was isolated to determine transcript levels for *Cxcr5*, *Il6ra*, *Tnfsf8,* and *Sh2d1a*. Samples were normalized to *Rps18* as a control and the data are represented relative to the IL-15_TRANS_ (75 ng/ml) sample. (**b**) Primary CD4^+^ T cells were cultured as described as in “a”. RNA was isolated to determine transcript levels for *Tbx21*, *Ifng*, *Il12rb1*, and *Il12rb2*. Samples were normalized to *Rps18* as a control. Data are presented relative to the IL-15_TRANS_ (75 ng/ml) sample. Four (**a,b**) independent experiments were performed with the error bars representing SEM. *P < 0.05, **P < 0.01, ***P < 0.001 (unpaired Student’s *t*-test). (**c**) Cells were treated and harvested as in “a”. Following cell isolation, an immunoblot assay was performed to assess STAT4 activation levels. Total STAT4 is shown as a control. The image shown is representative of three independent experiments performed.

In our previous study, we found that the T_FH_-associated genes *Cxcr5*, *Il6ra*, and *Tnfsf8* are all direct targets of Blimp-1-mediated repression^36^. Therefore, Bcl-6 activates the T_FH_ gene profile, at least in part, through the repression of a second transcriptional repressor (Blimp-1). Given the upregulation of Blimp-1 in response to IL-15 signaling, we hypothesized that the trans-presentation of IL-15 may also lead to the repression of the T_FH_ gene program. To investigate this possibility, we examined the expression of the T_FH_-associated genes *Cxcr5*, *Il6ra*, *Tnfsf8,* and *Sh2d1a* under different IL-15 signaling conditions. Indeed, IL-15 signaling was associated with decreased expression of all four genes, in both dose- and trans-presentation-dependent manners (Fig. 4a). However, under low IL-15 signaling conditions, the expression of each gene was significantly induced. Once again, treating the cells with the trans-presented form of IL-15, even at low concentrations, was sufficient to maintain inhibition of *Cxcr5*, *Il6ra*, *Tnfsf8,* and *Sh2d1a* expression, most likely due to the increase in Blimp-1 expression (Fig. 3). Interestingly, not all T_FH_-associated genes responded similarly to IL-15 signaling. For example, the expression of *Pdcd1*, *Icos*, and *Il21* did not appear to be augmented by changes to IL-15 concentration or trans-presentation (Supplementary Fig. S2). These data strongly suggest that IL-15 signaling is not a comprehensive regulator of the entire T_FH_ gene program. This is, perhaps, not surprising, as it is well-established that there are required roles for B cell interactions, as well as pro-T_FH_ cytokines such as IL-6 and IL-21, in the full commitment to the T_FH_ cell fate^17^. Thus, it is likely that the *ex vivo* setting lacks at least some of the necessary environmental factors required to promote the induction of a complete T_FH_ gene profile.

As our data suggest that enhanced IL-15 signaling is capable of maintaining T_FH_ gene repression even in the absence of IL-2, we hypothesized that trans-presented IL-15 may also be sufficient to promote the expression of T_H_1-associated genes. Indeed, treatment with trans-presented IL-15 resulted in increased expression of both the T_H_1 lineage-defining transcription factor *Tbx21*, and canonical cytokine *Ifng*, when compared to IL-15 treatment alone (Fig. 4b). Furthermore, cells that were exposed to trans-presented IL-15 expressed higher levels of both *Il12rb1* and *Il12rb2* compared to cells that received IL-15 alone (Fig. 4b). Consistent with the observed increase in *Il12rb1* and *Il12rb2* expression, we found that exposure of cells to trans-presented IL-15 also resulted in an increase in IL-12-dependent STAT4 activation (Fig. 4c). Taken together, these findings suggest that intracellular signaling induced by trans-presented IL-15 can promote additional cytokine-mediated responses critical for the promotion or repression of the T_H_1 and T_FH_ gene programs, respectively.

## Discussion

CD4^+^ T cell differentiation from the naïve cell state into any of several effector cell types is dependent upon the cytokine environment in which the cell is located during its activation. Until recently, it was believed that CD4^+^ T cell differentiation into an effector subset was a terminal event, but a significant body of work has established that differentiated CD4^+^ T cells are capable of modulating their effector functions in response to changes in the cytokine environment^15^,^38^,^39^,^45^,^46^. This revelation may be particularly impactful given the use of cytokines and cytokine neutralizing antibodies as approaches to regulate the immune response in a variety of immunotherapeutic strategies.

One such example is the therapeutic use of the cytokine IL-15, which is currently under evaluation in clinical trials for the treatment of a number of types of cancer^32^,^33^,^34^. IL-15 may be an attractive alternative to IL-2-based therapies, as it is able to promote many of the same therapeutic effects due to similar downstream signaling pathways^47^. However, unlike IL-2, IL-15 does not support regulatory T cell populations or promote activation induced cell death (AICD), both of which may inhibit anti-cancer immune responses^48^. Although the effects of IL-15 signaling have been well characterized in CD8^+^ T and NK cell populations, the effect of IL-15 on the CD4^+^ T cell effector population has remained relatively unknown^49^. Thus, as the application of this promising cytokine increases, it will be crucial to understand its effects on these vital cell populations in a clinical setting.

Here, our data demonstrate that CD4^+^ T cells are capable of responding to IL-15 treatment, particularly when IL-15 is trans-presented in complex with the IL-15Rα subunit. Subsequently, IL-15 signaling and the concurrent activation of the transcription factor STAT5 are associated with the positive regulation of T_H_1 genes including *Tbx21*, *Ifng*, and *Prdm1*. Additionally, we observed increased expression of the IL-12 receptor subunits (*Il12rb1* and *Il12rb2*), as well as STAT4 activation, in response to IL-15 trans-presentation. This data is in agreement with previous work demonstrating a similar role for IL-2 in the positive regulation of the components of the IL-12R, and suggests that IL-2- and IL-15-signaling, in the context of trans-presentation, share at least some downstream effects^40^.

We also found that there is an IL-15 trans-presentation-dependent inhibitory effect upon the expression of genes associated with the T_FH_ cell fate, including the expression of the transcriptional repressor Bcl-6. Our findings indicate that there is an inverse relationship between STAT5 activation and Bcl-6 expression, hinting at a potential regulatory mechanism whereby STAT5 may directly repress Bcl-6. As these data are similar to previous studies examining the repressive effect of IL-2 signaling on the T_FH_ gene program, they suggest that the combined action of STAT5 and Blimp-1 may be a conserved regulatory mechanism utilized by IL-2 and IL-15 signaling to promote the T_H_1 cell fate^36^,^50^,^51^. Further investigation will be necessary to fully elucidate whether there are specific transcriptional mechanisms unique to IL-15 signaling that both inhibit and promote the expression of the T_FH_ and T_H_1 gene programs, respectively.

Despite the apparent antagonistic nature of Bcl-6 expression and IL-15 signaling, it is interesting to note that both factors have been shown to positively influence the formation of T cell memory. Bcl-6 expression has been linked to the promotion of the effector-to-memory cell transition in both CD4^+^ and CD8^+^ T cells, while IL-15 is critical for the homeostatic maintenance of memory cell populations^42^,^52^,^53^. Future work will be necessary to dissect how the findings presented in this study may translate into an increased understanding of the interplay between these intrinsic and extrinsic factors that govern memory cell fate.

Collectively, this study highlights a novel role for IL-15 signaling in modulating the gene expression profiles of CD4^+^ T helper cell populations. Consequently, these changes in gene expression dictate both the fate and function of the responding cells. As such, an increased understanding of the implications of cytokine signaling, including that of IL-15, on the plasticity of CD4^+^ T cell populations will be critical as these environmental modulators continue to be utilized in novel immunotherapeutic strategies.

## Methods

### Primary cells and cell culture

Primary CD4^+^ T cells were isolated from the spleens and lymph nodes of 5–8 week old C57BL/6 mice using the R&D Systems MagCellect CD4^+^ T cell Isolation Kit according to the manufacturer’s instructions. Cells were cultured in complete Iscove’s Modified Dulbecco’s medium [cIMDM; IMDM (12440053, Life Technologies), 10% FBS (26140079, Life Technologies), 1% Penicillin-Streptomycin (15140122, Life Technologies), 0.05% BME (BP176, Fisher Scientific)] and plated on αCD3 (BDB553057, Fisher Scientific) and αCD28 (BDB553294, Fisher Scientific) under T_H_1 polarizing conditions (IL-12, αIL-4, and IL-2) as described previously^36^,^54^. After three days (or five, as in Supplementary Fig. 1), cells were split and maintained for an additional two days in IL-12 and αIL-4 and either high (75 ng/ml) or low (15 ng/ml) concentrations of IL-15, or a stable complex of IL-15 and its receptor (referred to in this manuscript as trans-presented IL-15, or IL-15_TRANS_), as indicated. The Institutional Animal Care and Use Committee of Virginia Tech approved all experimentation involving the use of mice. All methods were performed in accordance with the approved guidelines.

### Generation of the IL-15/IL15Rα complex

A stable complex of IL-15/IL-15Rα was generated by combining soluble IL-15 (447-ML, R&D Systems) with a recombinant mouse IL-15Rα Fc chimera (551-MR, R&D Systems) at a ratio of 2:1. The combination was incubated at 37 °C for 45 minutes immediately prior to use, as described previously^29^,^41^.

### RNA isolation and qRT-PCR

Two days following IL-15 cytokine treatment, cells were harvested for each condition and RNA was isolated using the Nucleospin RNA II kit from Macherey-Nagel (740955.5) per the manufacturer’s protocol. cDNA was generated using the Superscript First Strand Synthesis Kit (11904018, Life Technologies). Triplicate qRT-PCR reactions using 10–20 ng of template were performed in 20μL reactions with gene-specific primers (Supplementary Table S1) and iTaq Universal SYBR Green Supermix (172-5124, Bio-Rad). All samples were normalized to the ribosomal protein S18 (*Rps18*) control. For IL-2-treated samples, expression levels were normalized to the high IL-2-treated sample. For IL-15-treated samples, expression levels were normalized to the high IL-15_TRANS_-treated sample.

### Immunoblot analysis

To examine the effect of IL-15 and IL-15_TRANS_ at the protein level, primary T cells for each condition were harvested 2 days following IL-2, IL-15, or IL-15_TRANS_ treatment. Antibodies used were as follows: pSTAT5 (611964, BD Biosciences), STAT5 (sc-482X, Santa Cruz), Bcl-6 (561520, BD Biosciences), pSTAT4 (5267S, Cell Signaling), STAT4 (sc-486X, Santa Cruz), Blimp-1 (A01647-40, Genscript), GAPDH (sc-25778, Santa Cruz), β-Actin (A00730-40, Genscript). Further details regarding the clone and/or dilution of antibodies used can be found in Supplementary Table S2).

### Statistical analysis

All data represent at least three independent experiments. Error bars represent the standard error of the mean (SEM). For statistical analysis, unpaired *t* tests were performed using GraphPad Prism online software. *P* values < 0.05 were considered statistically significant.

## Additional Information

**How to cite this article**: Cooley, I. D. *et al.* Trans-presentation of IL-15 modulates STAT5 activation and Bcl-6 expression in T_H_1 cells. *Sci. Rep.* **5**, 15722; doi: 10.1038/srep15722 (2015).

## Supplementary Material

Supplementary Information

## References

[b1] Crotty S. Follicular Helper CD4 T Cells (T(FH)). Annu Rev Immunol 29, 621–663 (2011). 21314428 10.1146/annurev-immunol-031210-101400

[b2] O’Shea J. J. & Paul W. E. Mechanisms underlying lineage commitment and plasticity of helper CD4+ T cells. Science 327, 1098–1102 (2010). 20185720 10.1126/science.1178334 PMC2997673

[b3] Reinhardt R. L., Kang S. J., Liang H. E. & Locksley R. M. T helper cell effector fates—who, how and where? Curr Opin Immunol 18, 271–277 (2006). 16617008 10.1016/j.coi.2006.03.003

[b4] Zhu J. & Paul W. E. CD4 T cells: fates, functions, and faults. Blood 112, 1557–1569 (2008). 18725574 10.1182/blood-2008-05-078154 PMC2518872

[b5] Rudensky A. Y. Regulatory T cells and Foxp3. Immunol Rev 241, 260–268 (2011). 21488902 10.1111/j.1600-065X.2011.01018.x PMC3077798

[b6] Fontenot J. D., Gavin M. A. & Rudensky A. Y. Foxp3 programs the development and function of CD4+ CD25+ regulatory T cells. Nat Immunol 4, 330–336 (2003). 12612578 10.1038/ni904

[b7] Hori S., Nomura T. & Sakaguchi S. Control of regulatory T cell development by the transcription factor Foxp3. Science 299, 1057–1061 (2003). 12522256 10.1126/science.1079490

[b8] Ivanov II *et al.* The orphan nuclear receptor RORgammat directs the differentiation program of proinflammatory IL-17+ T helper cells. Cell 126, 1121–1133 (2006). 16990136 10.1016/j.cell.2006.07.035

[b9] Johnston R. J. *et al.* Bcl6 and Blimp-1 are reciprocal and antagonistic regulators of T follicular helper cell differentiation. Science 325, 1006–1010 (2009). 19608860 10.1126/science.1175870 PMC2766560

[b10] Nurieva R. I. *et al.* Bcl6 mediates the development of T follicular helper cells. Science 325, 1001–1005 (2009). 19628815 10.1126/science.1176676 PMC2857334

[b11] Szabo S. J. *et al.* A novel transcription factor, T-bet, directs Th1 lineage commitment. Cell 100, 655–669 (2000). 10761931 10.1016/s0092-8674(00)80702-3

[b12] Yu D. *et al.* The transcriptional repressor Bcl-6 directs T follicular helper cell lineage commitment. Immunity 31, 457–468 (2009). 19631565 10.1016/j.immuni.2009.07.002

[b13] Bonelli M. *et al.* Helper T cell plasticity: impact of extrinsic and intrinsic signals on transcriptomes and epigenomes. Curr Top Microbiol Immunol 381, 279–326 (2014). 24831346 10.1007/82_2014_371 PMC4200396

[b14] Geginat J. *et al.* Plasticity of human CD4 T cell subsets. Front Immunol 5, 630 (2014). 25566245 10.3389/fimmu.2014.00630 PMC4267263

[b15] Zhou L., Chong M. M. & Littman D. R. Plasticity of CD4^+^ T cell lineage differentiation. Immunity 30, 646–655 (2009). 19464987 10.1016/j.immuni.2009.05.001

[b16] Oestreich K. J. & Weinmann A. S. Master regulators or lineage-specifying? Changing views on CD4(+) T cell transcription factors. Nat Rev Immunol 12, 799–804 (2012). 23059426 10.1038/nri3321 PMC3584691

[b17] Crotty S. T follicular helper cell differentiation, function, and roles in disease. Immunity 41, 529–542 (2014). 25367570 10.1016/j.immuni.2014.10.004 PMC4223692

[b18] Lazarevic V. & Glimcher L. H. T-bet in disease. Nat Immunol 12, 597–606 (2011). 21685955 10.1038/ni.2059 PMC6290474

[b19] Liao W., Lin J. X. & Leonard W. J. Interleukin-2 at the crossroads of effector responses, tolerance, and immunotherapy. Immunity 38, 13–25 (2013). 23352221 10.1016/j.immuni.2013.01.004 PMC3610532

[b20] Slifka M. K. & Amanna I. How advances in immunology provide insight into improving vaccine efficacy. Vaccine 32, 2948–2957 (2014). 24709587 10.1016/j.vaccine.2014.03.078 PMC4096845

[b21] Tangye S. G., Ma C. S., Brink R. & Deenick E. K. The good, the bad and the ugly—TFH cells in human health and disease. Nat Rev Immunol 13, 412–426 (2013). 23681096 10.1038/nri3447

[b22] Liao W., Lin J. X. & Leonard W. J. IL-2 family cytokines: new insights into the complex roles of IL-2 as a broad regulator of T helper cell differentiation. Curr Opin Immunol 23, 598–604 (2011). 21889323 10.1016/j.coi.2011.08.003 PMC3405730

[b23] Rochman Y., Spolski R. & Leonard W. J. New insights into the regulation of T cells by gamma(c) family cytokines. Nat Rev Immunol 9, 480–490 (2009). 19543225 10.1038/nri2580 PMC2814538

[b24] Castillo E. F. & Schluns K. S. Regulating the immune system via IL-15 transpresentation. Cytokine 59, 479–490 (2012). 22795955 10.1016/j.cyto.2012.06.017 PMC3422378

[b25] Dubois S., Mariner J., Waldmann T. A. & Tagaya Y. IL-15Ralpha recycles and presents IL-15 In trans to neighboring cells. Immunity 17, 537–547 (2002). 12433361 10.1016/s1074-7613(02)00429-6

[b26] Sandau M. M., Schluns K. S., Lefrancois L. & Jameson S. C. Cutting edge: transpresentation of IL-15 by bone marrow-derived cells necessitates expression of IL-15 and IL-15R alpha by the same cells. J Immunol 173, 6537–6541 (2004). 15557143 10.4049/jimmunol.173.11.6537

[b27] Stonier S. W. & Schluns K. S. Trans-presentation: a novel mechanism regulating IL-15 delivery and responses. Immunol Lett 127, 85–92 (2010). 19818367 10.1016/j.imlet.2009.09.009 PMC2808451

[b28] Schluns K. S., Klonowski K. D. & Lefrancois L. Transregulation of memory CD8 T-cell proliferation by IL-15Ralpha+ bone marrow-derived cells. Blood 103, 988–994 (2004). 14512307 10.1182/blood-2003-08-2814

[b29] Rubinstein M. P. *et al.* Converting IL-15 to a superagonist by binding to soluble IL-15R{alpha}. Proc Natl Acad Sci USA 103, 9166–9171 (2006). 16757567 10.1073/pnas.0600240103 PMC1482584

[b30] Hanick N. A. *et al.* Elucidation of the interleukin-15 binding site on its alpha receptor by NMR. Biochemistry 46, 9453–9461 (2007). 17655329 10.1021/bi700652f

[b31] Mortier E., Bernard J., Plet A. & Jacques Y. Natural, proteolytic release of a soluble form of human IL-15 receptor alpha-chain that behaves as a specific, high affinity IL-15 antagonist. J Immunol 173, 1681–1688 (2004). 15265897 10.4049/jimmunol.173.3.1681

[b32] Conlon K. C. *et al.* Redistribution, hyperproliferation, activation of natural killer cells and CD8 T cells, and cytokine production during first-in-human clinical trial of recombinant human interleukin-15 in patients with cancer. J Clin Oncol 33, 74–82 (2015). 25403209 10.1200/JCO.2014.57.3329 PMC4268254

[b33] Wu J. IL-15 Agonists: The Cancer Cure Cytokine. J Mol Genet Med 7, 85 (2013). 24587813 10.4172/1747-0862.1000085 PMC3938108

[b34] Waldmann T. A. *et al.* Phase 1 trial of IL-15 trans presentation blockade using humanized Mikbeta1 mAb in patients with T-cell large granular lymphocytic leukemia. Blood 121, 476–484 (2013). 23212516 10.1182/blood-2012-08-450585 PMC3548167

[b35] Oestreich K. J., Huang A. C. & Weinmann A. S. The lineage-defining factors T-bet and Bcl-6 collaborate to regulate Th1 gene expression patterns. J Exp Med 208, 1001–1013 (2011). 21518797 10.1084/jem.20102144 PMC3092354

[b36] Oestreich K. J., Mohn S. E. & Weinmann A. S. Molecular mechanisms that control the expression and activity of Bcl-6 in TH1 cells to regulate flexibility with a TFH-like gene profile. Nat Immunol 13, 405–411 (2012). 22406686 10.1038/ni.2242 PMC3561768

[b37] Basu R., Hatton R. D. & Weaver C. T. The Th17 family: flexibility follows function. Immunol Rev 252, 89–103 (2013). 23405897 10.1111/imr.12035 PMC3607325

[b38] Murphy K. M. & Stockinger B. Effector T cell plasticity: flexibility in the face of changing circumstances. Nat Immunol 11, 674–680 (2010). 20644573 10.1038/ni.1899 PMC3249647

[b39] Zhu J. & Paul W. E. Heterogeneity and plasticity of T helper cells. Cell Res 20, 4–12 (2010). 20010916 10.1038/cr.2009.138 PMC3494736

[b40] Liao W., Lin J. X., Wang L., Li P. & Leonard W. J. Modulation of cytokine receptors by IL-2 broadly regulates differentiation into helper T cell lineages. Nat Immunol 12, 551–559 (2011). 21516110 10.1038/ni.2030 PMC3304099

[b41] Stoklasek T. A., Schluns K. S. & Lefrancois L. Combined IL-15/IL-15Ralpha immunotherapy maximizes IL-15 activity *in vivo*. J Immunol 177, 6072–6080 (2006). 17056533 10.4049/jimmunol.177.9.6072 PMC2847275

[b42] Crotty S., Johnston R. J. & Schoenberger S. P. Effectors and memories: Bcl-6 and Blimp-1 in T and B lymphocyte differentiation. Nat Immunol 11, 114–120 (2010). 20084069 10.1038/ni.1837 PMC2864556

[b43] Martins G. & Calame K. Regulation and functions of Blimp-1 in T and B lymphocytes. Annu Rev Immunol 26, 133–169 (2008). 18370921 10.1146/annurev.immunol.26.021607.090241

[b44] Oestreich K. J. & Weinmann A. S. T-bet employs diverse regulatory mechanisms to repress transcription. Trends Immunol 33, 78–83 (2012). 22133865 10.1016/j.it.2011.10.005 PMC3273642

[b45] Hirahara K. *et al.* Helper T-cell differentiation and plasticity: insights from epigenetics. Immunology 134, 235–245 (2011). 21977994 10.1111/j.1365-2567.2011.03483.x PMC3209564

[b46] Nakayamada S., Takahashi H., Kanno Y. & O’Shea J. J. Helper T cell diversity and plasticity. Curr Opin Immunol 24, 297–302 (2012). 22341735 10.1016/j.coi.2012.01.014 PMC3383341

[b47] Sim G. C. & Radvanyi L. The IL-2 cytokine family in cancer immunotherapy. Cytokine Growth Factor Rev 25, 377–390 (2014). 25200249 10.1016/j.cytogfr.2014.07.018

[b48] Steel J. C., Waldmann T. A. & Morris J. C. Interleukin-15 biology and its therapeutic implications in cancer. Trends Pharmacol Sci 33, 35–41 (2012). 22032984 10.1016/j.tips.2011.09.004 PMC3327885

[b49] Mishra A., Sullivan L. & Caligiuri M. A. Molecular pathways: interleukin-15 signaling in health and in cancer. Clinical Cancer Res 20, 2044–2050 (2014). 24737791 10.1158/1078-0432.CCR-12-3603 PMC3989546

[b50] Johnston R. J., Choi Y. S., Diamond J. A., Yang J. A. & Crotty S. STAT5 is a potent negative regulator of TFH cell differentiation. J Exp Med 209, 243–250 (2012). 22271576 10.1084/jem.20111174 PMC3281266

[b51] Nurieva R. I. *et al.* STAT5 protein negatively regulates T follicular helper (Tfh) cell generation and function. J Biol Chem 287, 11234–11239 (2012). 22318729 10.1074/jbc.M111.324046 PMC3322890

[b52] Gasper D. J., Tejera M. M. & Suresh M. CD4 T-cell memory generation and maintenance. Crit Rev Immunol 34, 121–146 (2014). 24940912 10.1615/critrevimmunol.2014010373 PMC4062920

[b53] Kaech S. M. & Cui W. Transcriptional control of effector and memory CD8(+) T cell differentiation. Nat Rev Immunol 12, 749–761 (2012). 23080391 10.1038/nri3307 PMC4137483

[b54] Oestreich K. J. *et al.* Bcl-6 directly represses the gene program of the glycolysis pathway. Nat Immunol 15, 957–964 (2014). 25194422 10.1038/ni.2985 PMC4226759

