# Supplementary material for: Trans-presentation of IL-15 modulates STAT5 activation and Bcl-6 expression in TH1 cells
Source: Sci Rep. 2015 Oct 26;5:15722. doi: 10.1038/srep15722 (PMC4620557; doi:10.1038/srep15722)
Supplement: Supplementary Information [file srep15722-s1.pdf]

Supplementary Information

Trans-presentation of IL-15 modulates STAT5 activation and Bcl-6 expression in T_H1 cells

Ian D. Cooley, Kaitlin A. Read & Kenneth J. Oestreich

Supplementary Figure S1

Supplementary Figure S2

Supplementary Table S1

Supplementary Table S2

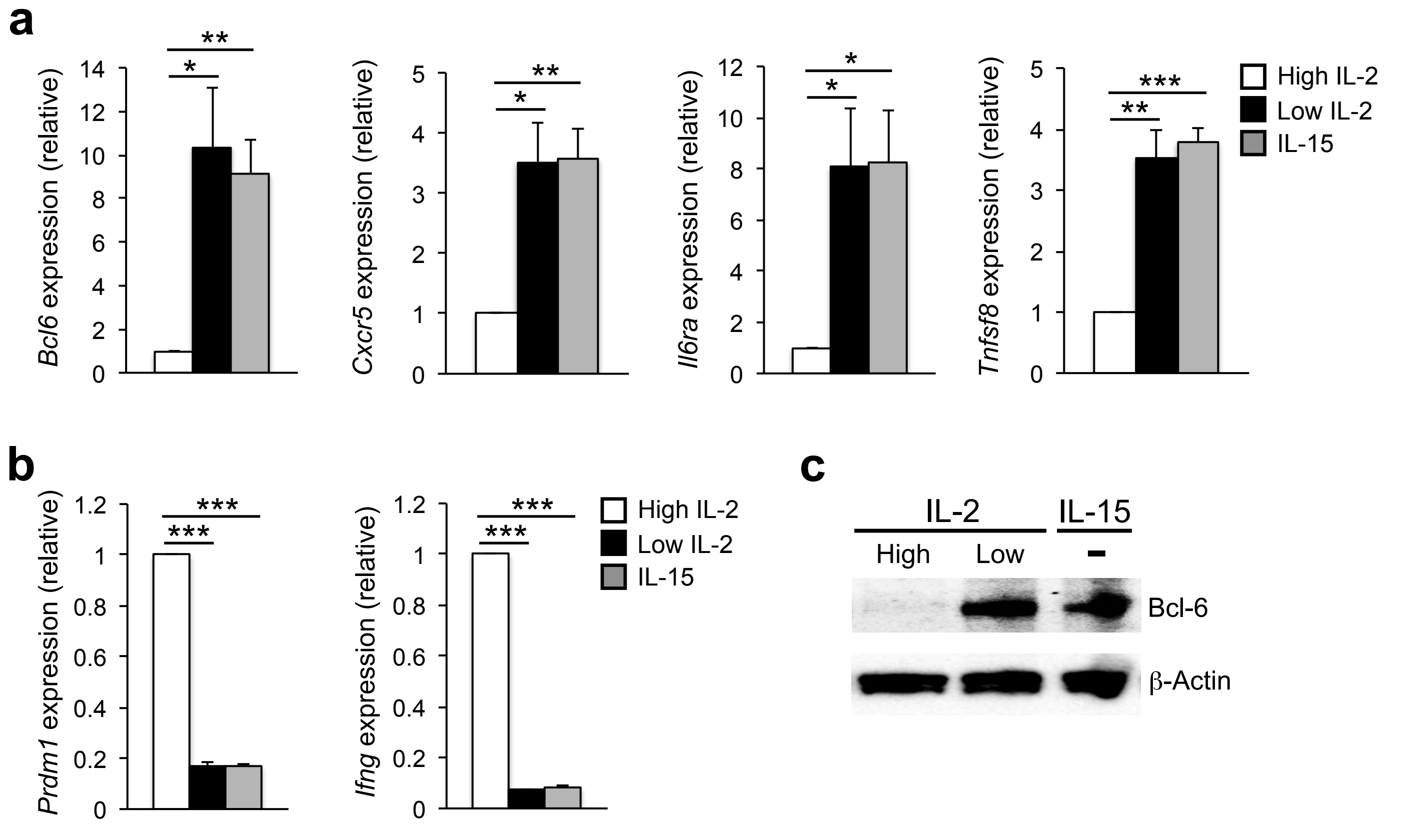

Supplementary Figure S1. T_H1 cells maintain flexibility with a T_{FH}-like profile after extended culture in T_H1 conditions.

Primary $CD4^+$ T cells were isolated from C57BL/6 mice and stimulated on plate-bound αCD3 and αCD28 for 3 days under T_H1-polarizing conditions. Cells were then split and cultured for additional 2 days under T_H1-skewing conditions. On day 5, cells were split and cultured with either high IL-2 (250 U/ml), low IL-2 (10 U/ml), or IL-15 (15 ng/ml). (**a**,**b**) Two days following cytokine treatment, RNA was isolated to determine transcript levels for the indicated T_{FH} and T_H1-associated genes. Samples were normalized to *Rps18* as a control. Data are presented relative to the high IL-2-treated sample. Three independent experiments were performed with the error bars representing SEM. *$P < 0.05$, **$P < 0.01$, ***$P < 0.001$ (unpaired Student's *t*-test). (**c**) An immunoblot analysis was performed to examine the effect of IL-2 and IL-15 signaling on Bcl-6 expression. β-Actin was monitored to ensure equal protein loading. For (**c**), the image is representative of three independent experiments.

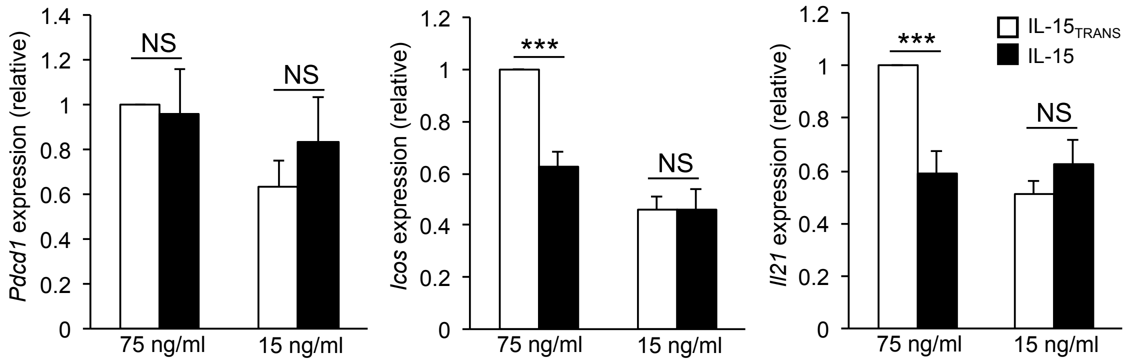

Supplementary Figure 2. Expression of notable T_{FH} genes is independent of IL-15 signaling. Primary $CD4^+$ T cells were cultured as described in Figure 2. RNA was isolated to determine transcript levels for *Pdcd1*, *Icos*, and *Il21*. Samples were normalized to *Rps18* as a control and the data are represented relative to the IL-15_{TRANS} (75 ng/ml) sample. Six independent experiments were performed with the error bars representing SEM. ****P* < 0.001 (unpaired Student's *t*-test).

Supplementary Table S1. Primer sequences

qRT-PCR primer sequences

Gene	Species	Sequence (5' to 3')
Rps18	mouse	fwd: GGAGAACTCACGGAGGATGAG<br>rev: CGCAGCTTGTTGTCTAGACCG
Bcl6	mouse	fwd: CCAACCTGAAGACCCACACTC<br>rev: GCGCAGATGGCTCTTCAGAGTC
Cxcr5	mouse	fwd: GTACCTAGCCATCGTCCATGC<br>rev: GTGCACTGTGGTAAGGAGTCG
IL6ra	mouse	fwd: CCACATAGTGTCACTGTGCG<br>rev: GGTATCGAAGCTGGAACTGC
Tnfsf8	mouse	fwd: GCAGCTACTTCTACCTCAGCAC<br>rev: GTGCCATCTTCGTTCCATGACAG
Prdm1	mouse	fwd: CTTGTGTGGTATTGTCGGGAC<br>rev: CACGCTGTACTCTCTCTTGG
Ifng	mouse	fwd: CTACCTTCTTCAGCAACAGC<br>rev: GCTCATTGAATGCTTGGCGC
Sh2d1a	mouse	fwd: CTGGATGGAAGCTATCTGCTG<br>rev: CAGGTGCTGTCTCGGCACTCC
IL12rb1	mouse	fwd: CACGACTCGGCTCCTCATGGAC<br>rev: TCTCAACGCAGCAGCCATCACC
IL12rb2	mouse	fwd: CTTGGACGGCATCAGTGTCTGC<br>rev: GACCTGGTGAGGAGCCAGCAAC
Tbx21	mouse	fwd: GTCCAAGTTCAACCAGCACC<br>rev: GTTGGTGAGCTTTAGCTTCC
Pdcd1	mouse	fwd: CGTCCCTCAGTCAAGAGGAG<br>rev: GTCCCTAGAAGTGCCCAACA
Icos	mouse	fwd: CTCACCAAGACCAAGGGAAGC<br>rev: CCACAACGAAAGCTGCACACC
IL21	mouse	fwd: TGGATCCTGAACTTCTATCAGCTCC<br>rev: AGGCAGCCTCCTCCTGAGC

Supplementary Table S2. Antibodies

Primary antibodies

Antibody	Dilution/Concentration	Species	Clone	Catalog#	Source
β-actin:HRP	1:10,000 (WB)	mouse	2D1D10	A00730	Genscript
Bcl-6	1:500 (WB)	mouse	K112-91	561520	BD Biosciences
Blimp-1	1:500 (WB)	rabbit		A01647	Genscript
STAT5 (C-17)	1:5000 (WB)	rabbit		sc-835X	Santa Cruz
pSTAT5 (pY694)	1:5000 (WB)	mouse	47/Stat5(pY694)	611964	BD Biosciences
GAPDH (FL-335)	1:2500 (WB)	rabbit		sc-25778	Santa Cruz
STAT4 (C-20)	1:2500 (WB)	rabbit		sc-486	Santa Cruz
pSTAT4 (pTyr693)	1:1000 (WB)	rabbit		5267	Cell Signalling
